# An MRI radiomics-based model for the prediction of invasion of the lymphovascular space in patients with cervical cancer

**DOI:** 10.3389/fonc.2024.1394427

**Published:** 2024-07-05

**Authors:** Nan-Nan Ma, Tao Wang, Ya-Nan Lv, Shao-Dong Li

**Affiliations:** ^1^ Department of Medical Imaging, Xuzhou Clinical School of Xuzhou Medical University, Xuzhou, China; ^2^ Department of Radiology, Xuzhou Central Hospital, Xuzhou, China; ^3^ Department of Radiology, Xuzhou Universal Medical Imaging Diagnostic Center, Xuzhou, China; ^4^ Department of Radiology, Affiliated Hospital of Xuzhou Medical University, Xuzhou, China

**Keywords:** MRI, radiomics, lymphovascular space invasion, cervical cancer, prediction, model

## Abstract

**Background:**

Cervical cancer (CC) remains the second leading cause of cancer-related death in women, and the ability to accurately anticipate the presence or absence of lymphovascular space invasion (LVSI) is critical to maintaining optimal patient outcomes. The objective of this study was to establish and verify an MRI radiomics-based model to predict the status of LVSI in patients with operable CC.

**Methods:**

The current study performed a retrospective analysis, with 86 patients in the training cohort and 38 patients in the testing group, specifically focusing on patients with CC. The radiomics feature extraction process included ADC, T2WI-SPAIR, and T2WI sequences. The training group data were used for the initial radionics-based model building, and the model predictive performance was subsequently validated using data from patients recruited in the experimental group.

**Results:**

The development of the radiomics scoring model has been completed with 17 selected features. The study found several risk factors associated with LVSI. These risk factors included moderate tumor differentiation (P = 0.005), poor tumor differentiation (P = 0.001), and elevated combined sequence-based radiomics scores (P = 0.001). Radiomics scores based on predictive model, combined sequences, ADC, T2WI-SPAIR, and T2WI exhibited AUCs of 0.897, 0.839, 0.815, 0.698, and 0.739 in the training cohort, respectively, with corresponding testing cohort values of 0.833, 0.833, 0.683, 0.692, and 0.725. Excellent consistency was shown by the calibration curve analysis, which showed a higher degree of agreement between the actual and anticipated LVSI status. Moreover, the decision curve analysis outcomes demonstrated the medical application of this prediction model.

**Conclusion:**

This investigation indicated that the MRI radiomics model was successfully developed and validated to predict operable CC patient LVSI status, attaining high overall diagnostic accuracy. However, further external validation and more deeper analysis on a larger sample size are still needed.

## Introduction

At the global level, cervical cancer (CC) remains the second deadliest form of cancer among women (1), with an estimated 530,000 diagnoses and 250,000 deaths annually (2). Surprisingly, patients in underdeveloped countries account for almost 80% of CC-related mortality (2). Despite recent therapy advancements, long-term CC patient outcomes are poor (3).

The occurrence of lymph node (LN) metastasis (LNM) is strongly connected with the prognosis of CC patients (4–6), and evidence of lymphovascular space invasion (LVSI) is significantly related to the LNM status of a given page. In patients with early-stage CC without LNM, positive LVSI status implies the necessity for lymph node dissection (7). Postoperative radiotherapy is also recommended in some LVSI-positive CC cases as per the Sedlis criteria (8). The ability to preoperatively gauge the LVSI status of a given patient is thus vital to support therapeutic decision-making. However, only postoperative pathology investigations may now confirm the presence of LVSI. Magnetic resonance imaging (MRI) techniques commonly diagnose CC because they provide excellent soft tissue resolution. However, standard MRI scans are poorly suited for diagnosing patient LVSI status (9–11).

Radiomics provides an alternate method for characterizing microscopic, invisible features to the human eye by analyzing clinical images to obtain high-dimensional quantitative data (12). MRI radiomics approaches have been deployed to predict tumor staging, LNM status, and therapeutic efficacy in patients with CC (4, 13–15). Currently, there have been no MRI radiomics studies that have explored investigated the ability to predict the status of LVSI in cervical cancer patients before surgery.

In order to fill this knowledge gap, the current study was conducted to develop and validate an MRI radiomics-based model that can accurately predict the state of LVSI in patients with operable CC.

## Study methodology

### Study design

This retrospective study was approved by the Ethics Committee of Xuzhou Central Hospital, with the need for informed consent being waived by the ethics committee. The training group consisted of 86 consecutive patients with CC who underwent an MRI examination from June 2016 to June 2021. Additionally, a separate experimental group comprised 38 consecutive CC patients who underwent MRI evaluation between July 2021 and October 2022.

The inclusion criteria were set as follows: (1) patients who were preoperatively diagnosed with CC by hysteroscopy, (2) patients who had MRI scans performed within 7 days before surgical resection, (3) patients who underwent hysterectomy with pelvic LN dissection, and (4) the LVSI status confirmed by pathological examination. Patients were excluded from these analyses if: (1) their clinical data were not complete, (2) the quality of images is poor, (3) they were pregnant patients, (4) they showed any other comorbid malignancies, or (5) they had undergone preoperative chemotherapy or radiotherapy.

The collected data from all participants encompassed baseline age, BMI, tumor differentiation, 2018 FIGO clinical staging, depth of invasion, and levels of serum tumor biomarkers (such as carcinoembryonic antigen [CEA], alpha-fetoprotein [AFP], carbohydrate antigen 199 [CA199], and squamous cell carcinoma antigen [SCC]).

### MRI analyses

The MRI scanning procedure was conducted utilizing a 1.5T MRI apparatus manufactured by Philips, employing a body array coil known as Ingenia. Each patient underwent axial T2WI, fat suppression T2WI, axial diffusion-weighted imaging (DWI), sagittal T1WI, and sagittal T2WI sequence. The SPAIR sequences were employed to suppress fat. For further details concerning individual scanning protocols, see **Supplementary Table 1**.

### Feature extraction

**Figure 1:**
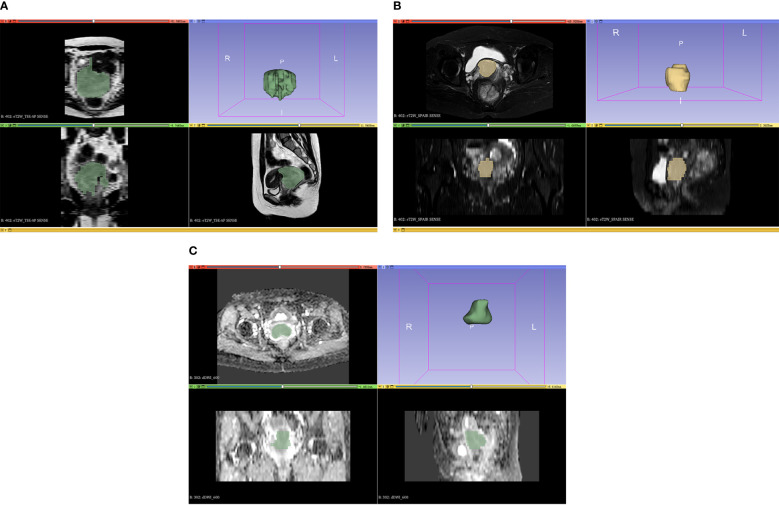
The figures of MRI segmentation on the sequences of **(A)** T2WI, **(B)** T2WI-SPAIR, and **(C)** ADC.

The axial ADC, axial T2WI-SPAIR, and Sagittal T2WI MRI results were analyzed with 3D Slicer (v 5.03) in which volumes of interest (VOIs) were defined by manually drawing the boundaries of target tumors (**Figure 1**). The VOIs were extracted by two radiologists, one with 10 years of experience and the other with 5 years of experience. The radiologists were uninformed about the pathological findings of the patients. Additionally, the 3D Slicer application was utilized for feature extraction, and the observer consistency was evaluated by considering both intra- and inter-class coefficient (ICC) values. Two radiologists independently segmented MRI images from a randomly selected training sample of 20 patients. In addition, one of these radiologists (Reader 1) repeated the segmentation of tumors from these same 20 patients 1-week after the initial segmentation. Features that demonstrated an ICC of at least ≥ 0.8 were deemed sufficiently reproducible and were kept for further examination. After that, Reader 1 segmented all remaining images.

### Feature selection

A variance threshold method was first used to determine whether the characteristics had a variance > 0.75 to select features. After that, the Selec-K-Best approach was used for the retained characteristics, retaining those with a P-value of > 0.05. Lastly, factors associated with LVSI status were selected using a LASSO regression model.

### Radiomics model formulation and testing

The selected characteristics were utilized to create a radiomics signature model, which allowed radiomics scores for each CC patient to be determined as follows:


$$\text{Rad}\_Score=Intercept+\sum_{i=1}^{n}\text{coefficients}\left[i\right]\times Feature\left[i\right]$$

Risk factors associated with the LVSI status of training cohort patients were selected via univariate and multivariate logistic regression approaches to enable the combined assessment of clinical features, serum biomarkers, and radiomics scores. These findings led to the development of a predictive nomogram, which was subsequently validated using the data from the testing cohort.

### Statistical analyses

Data were analyzed with SPSS 25.0 and R 4.1.2, comparing categorical data with χ^2^ tests or Fisher’s exact test. Normally (non-normally) distributed continuous data were compared with Student’s t-tests (Mann-Whitney U tests). Several logistic regression analyses were used to determine risk factors associated with LVSI status. The area under the curve (AUC) values for generated receiver operating characteristic (ROC) curves were compared using the DeLong test.

## Results

### Experimental group

The experimental group consisted of 86 patients diagnosed with cervical cancer, with 62 testing negative and 24 testing positive for LVSI. The data from these patients are summarized in **Table 1**. The age, BMI, FIGO staging, pathological CC type, and serum tumor marker concentration distributions were similar across these groups. The differentiation level (P = 0.001) and cervical stromal invasion depth (P = 0.007) did, however, differ significantly between LVSI-positive and LVSI-negative patients.

**Table 1:** Baseline data of the patients in the training and test groups.

	Training (n = 86)			Test (n = 38)			P value*
	LVSI (-)	LVSI (+)	P value	LVSI (-)	LVSI (+)	P value	
Patients’ number	62	24	–	30	8	–	–
Age (y)	57.5 (51.2, 64.8)	52 (47.2, 57.5)	0.068	53.4 ± 12.7	53.2 ± 6.8	0.964	0.214
BMI	22.6 ± 2.6	22.2 ± 2.7	0.541	23 ± 2.9	21.8 ± 1.5	0.109	0.612
Differentiation			0.001			0.137	0.508
Poor	6 (9.7%)	13 (54.2%)		3 (10%)	2 (25%)		
Moderate	26 (41.9%)	10 (41.7%)		13 (43.3%)	5 (62.5%)		
Well	30 (48.4%)	1 (4.2%)		14 (46.7%)	1 (12.5%)		
Tumor size			0.150			0.950	0.335
≤ 4 cm	39 (62.9%)	11 (45.8%)		16 (53.3%)	5 (62.5%)		
> 4 cm	23 (37.1%)	13 (54.2%)		14 (46.7%)	3 (37.5%)		
SCC			1.000			1.000	1.000
< 2.5 μg/L	22 (35.5%)	8 (33.3%)		10 (33.3%)	3 (37.5%)		
≥ 2.5 μg/L	40 (64.5%)	16 (66.7%)		20 (66.7%)	5 (62.5%)		
Ca199			0.496			1.000	1.000
< 37 U/L	52 (83.9%)	22 (91.7%)		25 (83.3%)	7 (87.5%)		
≥ 37 U/L	10 (16.1%)	2 (8.3%)		5 (16.7%)	1 (12.5%)		
AFP			0.057			0.309	1.000
< 7 μg/L	48 (77.4%)	23 (95.8%)		24 (80%)	8 (100%)		
≥ 7 μg/L	14 (22.6%)	1 (4.2%)		6 (20%)	0 (0%)		
CEA			0.203			0.587	0.573
< 5 μg/L	51 (82.3%)	16 (66.7%)		26 (86.7%)	6 (75%)		
≥ 5 μg/L	11 (17.7%)	8 (33.3%)		4 (13.3%)	2 (25%)		
FIGO stage			0.085			0.244	1.000
I	34 (54.8%)	8 (33.3%)		15 (50%)	3 (37.5%)		
II	27 (43.5%)	14 (58.3%)		15 (50%)	4 (50%)		
III	1 (1.6%)	2 (8.3%)		0 (0%)	1 (12.5%)		
Pathological types			0.965			0.159	0.891
Adenocarcinoma	16 (25.8%)	7 (29.2%)		9 (30%)	0 (0%)		
Others	46 (74.2%)	17 (70.8%)		21 (70%)	8 (100%)		

AFP, α-fetoprotein; BMI, body mass index; CEA, carcinoembryonicantigen; FIGO, International Federation of Gynecology and Obstetrics; LVSI, lymphovascular space invasion; SCC, squamouse cell carcinoma antigen.

*: P values between training and test groups.

### Feature selection

A total of 851 radiomics characteristics were extracted for each sequence (T2WI, T2WI-SPAIR, ADC). The development of radiomics scores was then based on combining these three sequences. See **Supplementary Table 2** for more information about the feature selection procedure. Ultimately, this strategy led to the selection of 17 features utilized for radiomics score calculations (**Supplementary Figure 1**). The corresponding coefficient values for these features and the combined sequence-based mean square errors are shown in **Supplementary Figure 2**.

### Predictive model development

In univariate and multivariate analyses, factors associated with LVSI included elevated combined sequence-based radiomics scores (P = 0.001), moderate differentiation (P = 0.005), and a poor differentiation (P = 0.001). The correlation between these parameters and the incidence of LVSI was validated by multivariate analyses (**Table 2**).

**Table 2:** Risk factors of the LVSI.

	Univariate analysis			Multivariate analysis		
	OR	95% CI	P value	OR	95% CI	P value
Age	0.97	0.93-1.01	0.2			
BMI	0.94	0.79-1.13	0.53			
Differentiation						
Poor	1			1		
Moderate	0.178	0.053-0.596	**0.005**	0.127	32.19-0	**0.005**
Well	0.015	0.002-0.141	**< 0.001**	0.022	12.679-0	**0.001**
Tumor size						
≤ 4 cm	1					
> 4 cm	2.004	0.772-5.203	0.153			
SCC	1.1	0.41-2.98	0.85			
Ca199	0.47	0.1-2.34	0.36			
AFP	0.15	0.02-1.2	0.07			
CEA	2.32	0.8-6.76	0.12			
FIGO stage						
I	1					
II	2.204	0.807-6.02	0.123			
III	8.500	0.683-105.752	0.096			
Pathological types	0.84	0.3-2.41	0.75			
Radiomics score of combined sequences	419.788	20.067-8781.676	**< 0.001**	310.646	0.581-166102.419	**0.001**

AFP, α-fetoprotein; BMI, body mass index; CEA, carcinoembryonicantigen; FIGO, International Federation of Gynecology and Obstetrics; LVSI, lymphovascular space invasion; SCC, squamouse cell carcinoma antigen. Bold values emphasis on the statistical significance (P < 0.05).

**Figure 2:**
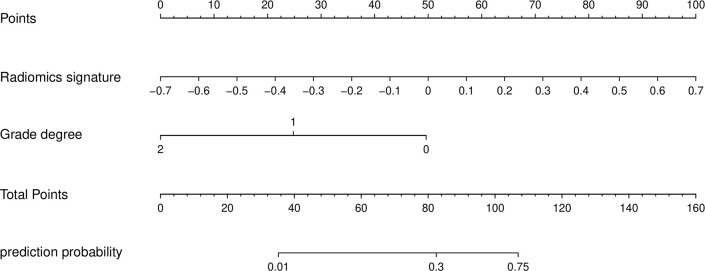
The nomogram of predictive model.

Next, a nomogram and predictive model were developed based on these results (**Figure 2**). The formula used to determine nomogram scores was as follows: score = 0.5202 - 1.9712×differentiation level (0: poor; 1: moderate; 2: well) ++ 5.6740 × combined sequence radiomics score.

**Figure 3:**
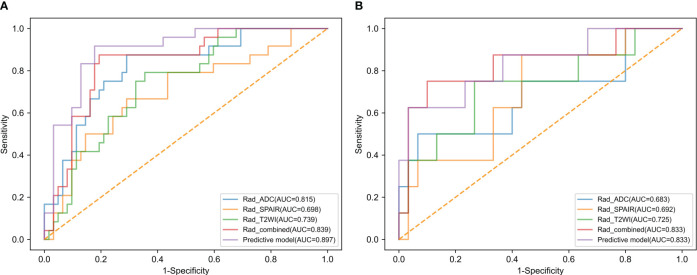
The ROC curves of radiomics score of T2WI, radiomics score of T2WI-SPAIR, radiomics score of ADC, radiomics score of combined sequences, and the predictive model in the **(A)** training and **(B)** test groups.

Analyzing CC patients in the training cohort, the prediction model showed an AUC of 0.897 and corresponding sensitivity and specificity values of 87.5% and 82.3% (**Figure 3A**, **Table 3**). This AUC value was higher than corresponding values based solely on radiomics scores based upon the T2WI (0.739), T2WI-SPAIR (0.698), ADC (0.815), and combined sequence (0.839) features (P = 0.006, 0.004, 0.067, and 0.144).

**Table 3:** Diagnostic performance of each parameter.

	Training group			Test group		
	Sensitivity	Specificity	AUC	Sensitivity	Specificity	AUC
Radiomics score of T2WI	70.8%	67.7%	0.739	75%	43.3%	0.725
Radiomics score of T2WI-SPAIR	79.2%	56.5%	0.698	87.5%	36.7%	0.692
Radiomics score of ADC	75%	77.4%	0.815	50%	63.3%	0.683
Radiomics score of combined sequences	87.5%	79%	0.839	87.5%	56.7%	0.833
Predictive model in this study	87.5%	82.3%	0.897	75.0%	76.7%	0.833

ADC, apparent diffusion coefficient; AUC, area under the curve.

### Testing cohort validation

The testing cohort consisted of 38 patients with CC, with 30 patients with LVSI-negative disease and 8 with LVSI-positive disease. Among the baseline parameters examined, the only significant difference observed between these two groups of patients was in the depth of cervical stromal invasion (**Table 1**). When the data from these patients were introduced into the developed predictive model, the respective AUC, sensitivity, and specificity values were 0.833, 75.0%, and 76.7% (**Figure 3B**, **Table 3**). While the predictive model-derived AUC value was higher than corresponding values for T2WI (0.725), T2WI-SPAIR (0.692), ADC (0.683), and combined sequence (0.833) based radiomics scores, these differences were not statistically significant (P = 0.293, 0.069, 0.137, and 1.000, respectively).

### Evaluation of predictive model clinical utility

**Figure 4:**
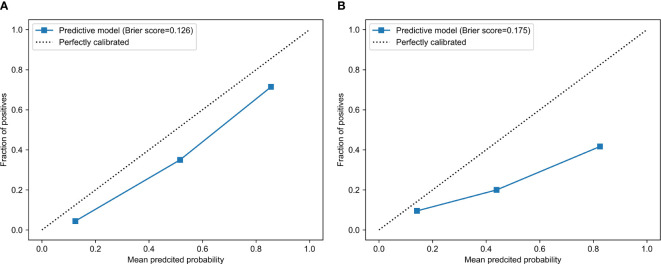
The calibration curves for **(A)** training and **(B)** test groups.

**Figure 5:**
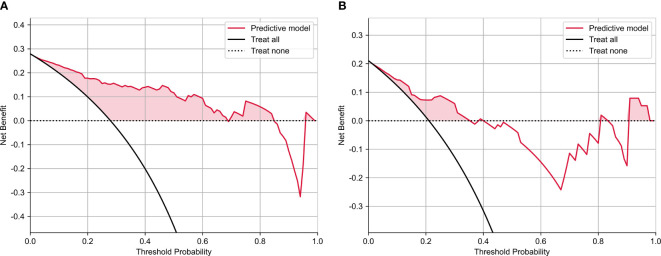
The decision curve analysis of nomograms of predictive model for **(A)** training and **(B)** test groups.

The calibration curve analysis demonstrated a high level of agreement between the model-predicted LVSI status of CC patients and their actual LVSI status in both the testing and training cohorts (**Figure 4**). Upon doing a decision curve analysis for this predictive model, it was found that the model produced significant net benefits in both the experimental and control groups. The risk thresholds for these groups were determined to be 0–0.84 and 0–0.36, respectively (**Figure 5**).

## Discussion

In this study, a model based on MRI radiomics was effectively created and validated as a predictive tool for the occurrence of lymphovascular space invasion (LVSI) in cervical cancer patients. A nomogram was ultimately established by combining MRI-based radiomics scores and relevant clinical features, and the resultant model exhibited high AUC values in training and testing cohorts consisting of individuals with CC (0.897 and 0.833, respectively). The data reported in this study suggest that the examined model can effectively guide healthcare decisions and treatment planning for patients with CC.

The ability to preoperatively assess LVSI status is critical in patients with early-stage CC because the presence or absence of LVSI determines the primary therapeutic strategy for individuals with clinical-stage IA disease (16–21). According to the National Comprehensive Cancer Network guidelines, surgery is often the main treatment for the early-stage CC (20). For the stage IA patients with LVSI(-), only cervical conization can be performed, thus avoiding radical hysterectomy, with the preservation of fertility (20). Furthermore, during the surgery for CC, LN dissection should be usually performed in order to test the LNM status (20, 21). LVSI was found to be a risk factor for LNM (19), Therefore, preoperative assessment of LVSI status may help to make the treatment decision. In addition, LVSI status was also found to be the risk factor for parametrial involvement and tumor recurrence (19). Therefore, preoperative evaluations of LVSI status is also important in the aspect of prognosis assessment.

Efficient evaluation of the appearance, anatomy, and molecular mobility of tumors can be achieved using conventional MRI scanning (18). However, since these scans cannot assess microscopic tumor pathology features, they are not well adapted for LVSI detection (17). Moreover, no direct MRI characteristic related to LVSI has been found.

Conventional pictures can be examined to derive radiomics features, which provide a means of evaluating tumors in greater depth and that correspond to a wide range of biological properties (17). Li et al. (19) previously developed an MRI radiomics-based model to predict CC patient LSVI status, but the AUC values for their training and testing cohorts were just 0.754 and 0.727. The AUC values in the prior study were likely lower than those in the present analysis since they only used T1WI sequences for feature extraction (19).

This study used a combination of many MRI sequences to extract radiomics features. The constructed predictive model yielded an AUC value of 0.897 when assessing patients in the training group. In addition, the sensitivity and specificity values were calculated to be 87.5% and 82.3%, respectively. The present study demonstrates the significance of a combined predictive radiomics scoring model, which surpasses the performance of radiomics scores produced from separate MRI sequences, as indicated by a more excellent AUC value. Utilizing multimodal MRI radiomics features can provide a more comprehensive understanding of the biological condition of a specific tumor compared to any single MRI sequence. The ultimate predictive model also included the differentiation level as the clinical characteristic strongly associated with the extent of CC malignancy. The model showed improved accuracy in predicting the LVSI status of CC patients by including these variables. The experimental group did not demonstrate a statistically significant increase in the AUC for the prediction model compared to those obtained from radiomics scores derived from the concatenated sequences, specifically ADC, T2WI, SPAIR, or T2WI. This can be attributed to the reduced sample size in this testing group.

This study has some limitations. Firstly, utilizing a retrospective patient group carries a significant potential for bias, underscoring the importance of doing prospective validation. Secondly, the limited number of patients recruited in the study restricts the statistical strength of these results. Because of this limitation, the detailed FIGO stages could not be divided and the FIGO stage was not associated with the LVSI status. Thirdly, the developed predictive model was based on a logistic regression analysis, and it may not be the most optimal predictive model given that machine learning models such as k-nearest neighbors, support vector machine, XG boost, random forest, or light GBM approaches were not employed. Lastly, LVSI status can be divided into the focal and diffused LVSI (19). However, we did not conduct any subgroup analysis for differentiating the focal and diffused LVSI due to the limited sample size. Further studies should be conducted to predict the diffused LVSI in CC patients.

## Conclusions

Overall, a radiomics-based model using MRI was successfully developed and utilized to predict the condition of LVSI in operable CC patients, showing a high level of diagnostic accuracy in all participants.

## Data availability statement

The original contributions presented in the study are included in the article/**Supplementary Material**. Further inquiries can be directed to the corresponding author.

## Ethics statement

The studies involving humans were approved by Ethics Committee of Xuzhou Central Hospital. The studies were conducted in accordance with the local legislation and institutional requirements. The ethics committee/institutional review board waived the requirement of written informed consent for participation from the participants or the participants’ legal guardians/next of kin because this is a retrospective study.

## Author contributions

N-NM: Data curation, Formal analysis, Writing – original draft. TW: Methodology, Software, Writing – review & editing. Y-NL: Methodology, Software, Writing – review & editing. S-DL: Conceptualization, Supervision, Writing – review & editing.

## Supplementary material

Supplementary Figure 1 Radiomics features and their coefficients.

Supplementary Figure 2 The **(A)** coefficient of each feature and **(B)** mean square error of the combined sequences.
