# Supplementary material for: An MRI radiomics-based model for the prediction of invasion of the lymphovascular space in patients with cervical cancer
Source: Front Oncol. 2024 Jul 5;14:1394427. doi: 10.3389/fonc.2024.1394427 (PMC11259963; doi:10.3389/fonc.2024.1394427)
Supplement: Supplementary file 1 [file Table_1.doc]

Table 1 The parameters of the MRI.

|  | TR/TE (ms) | FOV (mm) | Acquisition matrix | Slice thickness (mm) | Slice gap (mm) | NSA |
| --- | --- | --- | --- | --- | --- | --- |
| Sagittal T2WI | 3935/80 | 260×300 | 288×250 | 6 | 1 | 2 |
| Axial T2WI-SPAIR | 1450/100 | 210×210 | 252×200 | 3 | 1 | 2 |
| Axial DWI | 3040/65 | 375×300 | 124×101 | 6 | 1 | 7 |
| Sagittal T1WI | 400/10 | 210×210 | 210×165 | 3 | 1 | 1 |
| Axial T2WI | 5780/120 | 200×200 | 250×190 | 5 | 1 | 2 |

DWI: diffused-weight imaging; FOV: field of view; NSA: number of excites; TE: echo time; TR: repetition time.

Table 2 The radiomics features selection procedures.

| MRI sequences | Input features | Variance threshold (threshold=0.75) | Selec-K-Best (P < 0.05) | LASSO |
| --- | --- | --- | --- | --- |
| T2WI | 851 | 851 | 18 | 5 |
| T2WI-SPAIR | 851 | 851 | 4 | 3 |
| ADC | 851 | 851 | 51 | 8 |
| Combined | 2553 | 2553 | 73 | 17 |

ADC: apparent diffusion coefficient; LASSO: least absolute shrinkage and selection operator.
