# Supplementary figures and images for: An MRI radiomics-based model for the prediction of invasion of the lymphovascular space in patients with cervical cancer
Source: Front Oncol. 2024 Jul 5;14:1394427. doi: 10.3389/fonc.2024.1394427 (PMC11259963; doi:10.3389/fonc.2024.1394427)

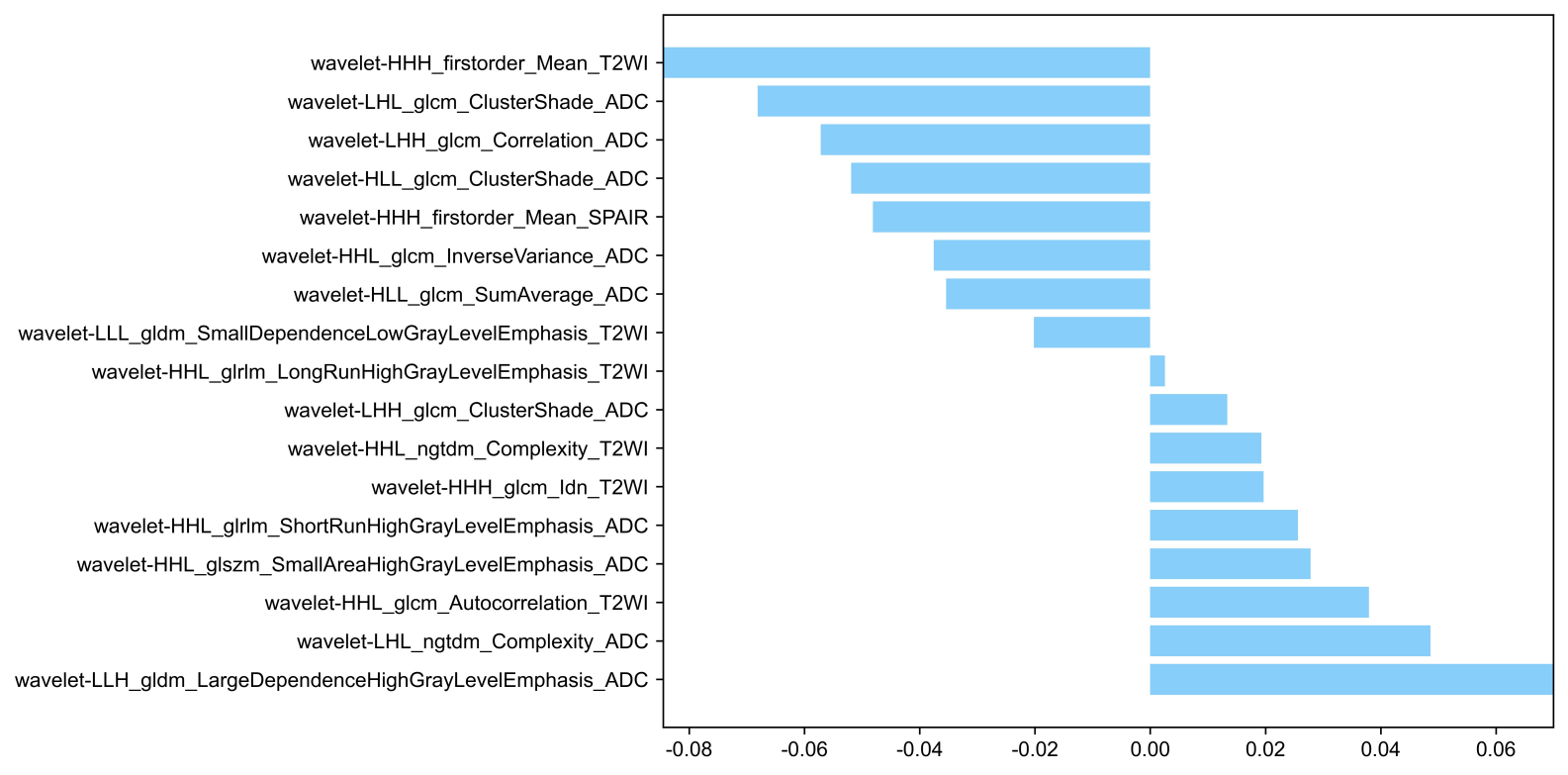

Supplement: Supplementary Figure 1 — Radiomics features and their coefficients. [file Image_1.png]

A
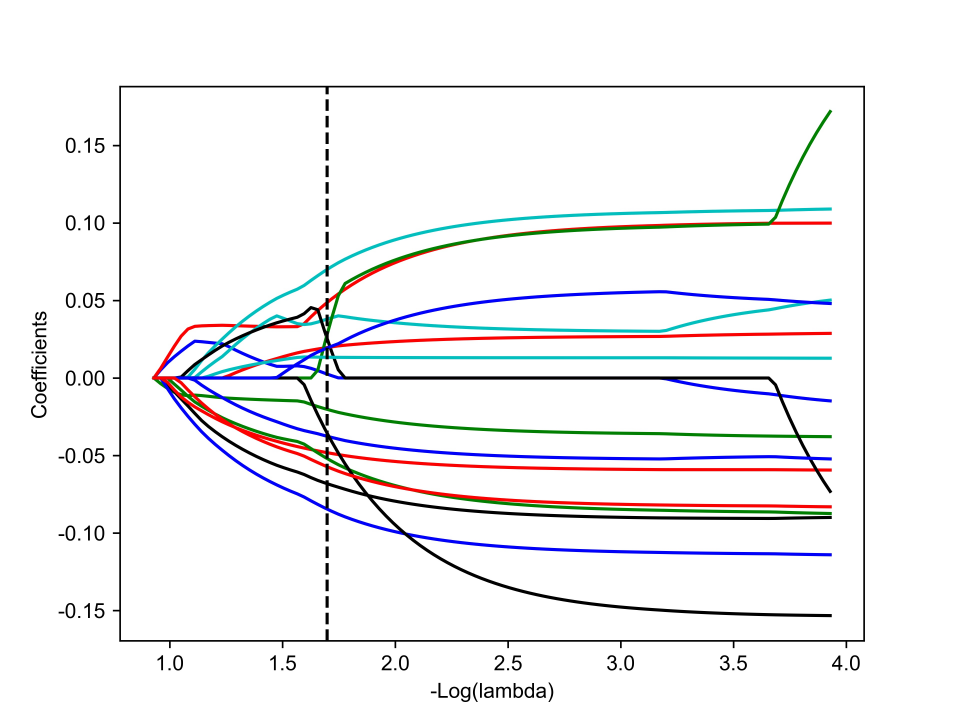


B
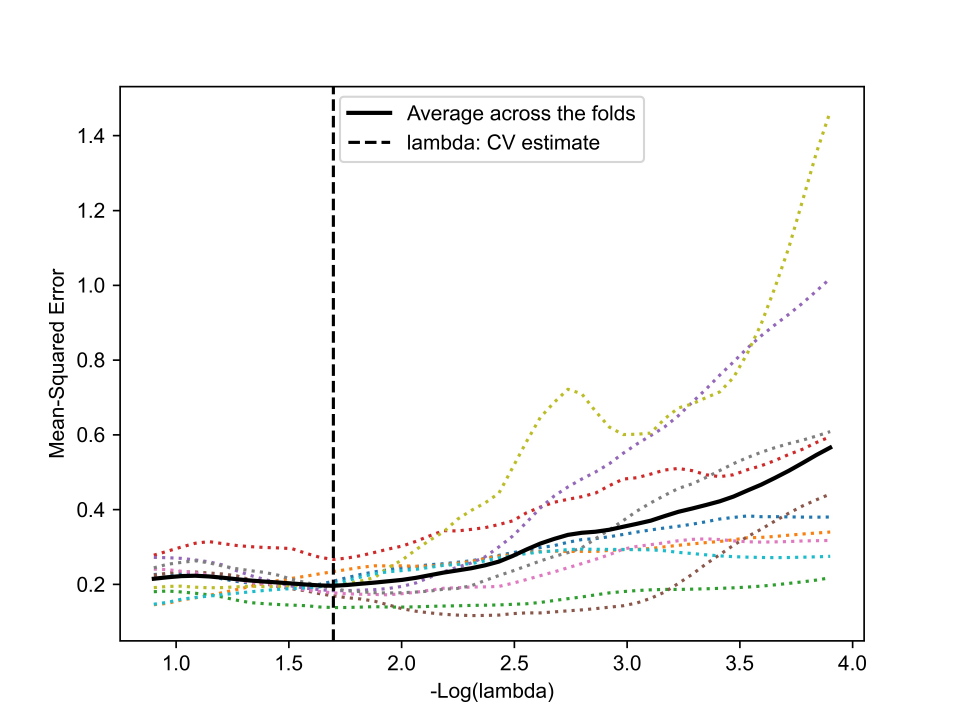

Supplement: Supplementary Figure 2 — The (A) coefficient of each feature and (B) mean square error of the combined sequences. [file DataSheet_1.doc]
